# Metabolomic Signatures of MASLD Identified by the Fatty Liver Index Reveal Gamma-Glutamyl Cycle Disruption and Lipid Remodeling

**DOI:** 10.3390/metabo15110687

**Published:** 2025-10-23

**Authors:** Khaled Naja, Najeha Anwardeen, Mohamed A. Elrayess

**Affiliations:** 1 Biomedical Research Center, QU Health, Qatar University, Doha P.O. Box 2713, Qatar; khaled.naja@qu.edu.qa (K.N.); n.anwardeen@qu.edu.qa (N.A.); 2 College of Medicine, QU Health, Qatar University, Doha P.O Box 2713, Qatar

**Keywords:** MASLD, FLI, metabolomics, gamma-glutamyl cycle, plasmalogens

## Abstract

Background/Objectives: Metabolic dysfunction-associated steatotic liver disease (MASLD) is the most prevalent chronic liver disorder worldwide and a key driver of cardiometabolic complications. Despite its growing burden, the underlying metabolic perturbations remain incompletely understood. The Fatty Liver Index (FLI) provides a validated non-invasive tool for stratifying MASLD in large-scale and clinical studies. Methods: This study utilized data from the Qatar Biobank, applying strict exclusion criteria and propensity score matching, to select 110 adults stratified by FLI into the MASLD group (≥60, *n* = 55) and the control group (<30, *n* = 55) with balanced age, sex, and BMI. Untargeted serum metabolomics was performed. Differential metabolite profiles were identified using linear regression adjusted for covariates and validated by multivariate modeling. Functional enrichment analyses were conducted to highlight perturbed metabolic pathways. Results: Metabolomic profiling revealed distinct metabolic signatures: the MASLD group was characterized by elevated glutamate and phospholipids, while the control group showed enrichment of gamma-glutamyl amino acids, plasmalogens, and sphingomyelins. Conclusions: This contrasting pattern reflects disruption of the gamma-glutamyl cycle and consistent depletion of antioxidant plasmalogen species, suggesting impaired redox homeostasis and lipid remodeling as hallmarks of MASLD pathogenesis. These findings provide a foundation for future research into targeted metabolic biomarkers and therapeutic strategies. Longitudinal and mechanistic studies are warranted to determine causal relationships and clinical utility.

## 1. Introduction

Metabolic dysfunction-associated steatotic liver disease (MASLD), superseding the former non-alcoholic fatty liver disease (NAFLD) terminology [1], reflects updated international expert consensus emphasizing metabolic dysfunction as the primary driver of liver steatosis and its clinical consequences. This global consensus synthesizes MASLD guidelines through early 2025 and defines diagnostic criteria and pathophysiological mechanisms, including insulin resistance, lipid dysregulation, and oxidative stress, and highlights approved therapeutic strategies [2,3]. MASLD has rapidly become the most common form of chronic liver disease, positioning it as the primary contributor to liver-related complications and mortality [4]. MASLD has been estimated to affect 30% of the adult population worldwide [5,6]. MASLD is driven by metabolic dysregulation, where insulin resistance leads to fat buildup in liver cells by increasing fatty acid delivery and reducing their breakdown. This fat accumulation can cause inflammation and liver damage, progressing from simple steatosis to steatohepatitis, fibrosis, and potentially cirrhosis or liver cancer [7]. The Fatty Liver Index (FLI), a non-invasive algorithm developed in 2006 by Bedogni and colleagues [8], has become a widely used and essential tool for the diagnosis and management of MASLD, particularly in large population studies and primary care settings [9]. FLI is superior to many other non-invasive tests because it incorporates serum triglycerides and measures of central obesity (waist circumference and BMI), capturing both fat accumulation and associated metabolic dysfunction [10].

Metabolomics plays a crucial role in understanding and managing MASLD by providing detailed insights into the metabolic changes underlying the disease. MASLD is a metabolic disorder, and metabolomics helps disclose the complex metabolic alterations involved in its development and progression. Previous metabolomic studies [11,12] have identified distinct circulating signatures, including specific dysregulations in phospholipids, acylcarnitines, bile acids, and amino acids, which are associated with the severity of steatosis, inflammation, and fibrosis. However, gaps remain in validating these biomarkers across diverse populations and in establishing standardized metabolomic panels for clinical use. Therefore, further studies are essential to address this metabolic complexity, improve diagnostic precision, and facilitate the development of targeted therapies, providing a strong rationale for continued investigation in this field.

This study aims to comprehensively compare the serum metabolomes of individuals with and without MASLD, classified using the validated FLI. By integrating differential metabolite profiling with pathway analysis, we seek to elucidate the biochemical mechanisms underlying MASLD pathogenesis. Our goal is to identify key metabolic alterations that can serve as early biomarkers, improve risk stratification, and reveal novel targets for personalized therapeutic interventions.

## 2. Methods

### 2.1. Data Source and Study Participants

This study utilized data from the Qatar Biobank (QBB), which includes detailed information on Qatari nationals and long-term residents [13]. The data collection included a comprehensive socio-demographic questionnaire and various clinical parameters [14]. All these measurements were performed at the Hamad Medical Corporation’s central laboratory, which is certified by the College of American Pathologists. Additionally, the dataset included information on medication usage [15], medical history, and a metabolomics profile covering more than 1000 metabolites using the Metabolon platform [16]. This research was approved by the Qatar Biobank’s institutional review boards (QF-QBB-RES-ACC-00178).

To minimize metabolic confounding and ensure a well-defined study population, individuals were excluded from the initial cohort of 2998 participants if they had T2D (to reduce metabolic variability), chronic liver diseases such as viral or autoimmune hepatitis (confirmed by clinical records), use of hepatotoxic or lipid-modifying medications (to avoid drug-induced metabolic alterations), renal insufficiency, history of major cardiovascular events, pregnancy or breastfeeding, or missing key data including gamma-glutamyl transferase (GGT), triglycerides (TG), body mass index (BMI), or waist circumference (WC).

The Fatty Liver Index was calculated using the following validated formula:

$$\mathrm{FLI}\;=\;\frac{e^{n}}{1+\;e^{n}}\times 100$$

where $n=0.953\times \ln\left(TG\right)+0.139\times BMI+0.718\times ln(GGT)+0.053\times WC-15.745$

The resulting FLI score ranges from 0 to 100, estimating the probability of hepatic steatosis. Participants were categorized as follows: FLI < 30 as the control group, FLI ≥ 60 as the fatty liver group [8,17], and those with FLI 30–59 were considered in the gray zone and were also excluded from the study to maximize contrast between metabolic profiles. To further reduce bias and ensure covariate balance, Propensity Score Matching was performed between the control and fatty liver groups. Matching covariates included age, sex, and BMI. Participants were matched 1:1 without replacement using nearest-neighbor matching with a caliper width of 0.2 of the standard deviation of the logit of the propensity score. Matching covariates included age, gender, and BMI, which are established confounders in metabolic studies. The quality of matching was assessed using standardized mean differences before and after matching, with all post-matching values of <0.1 indicating good covariate balance. Additionally, group-wise comparisons were used to verify that no significant group differences remained after matching. This procedure resulted in 55 matched participants in each group with balanced baseline characteristics.

### 2.2. Metabolomics

All participant serum samples were subjected to untargeted metabolomics using established protocols by Metabolon [18]. Metabolite measurement was performed using a Thermo Scientific Q-Exactive high-resolution/accurate mass spectrometer (Thermo Fisher Scientific, Inc., Waltham, MA, USA) interfaced with a heated electrospray ionization (HESI-II) source and Orbitrap mass analyzer operated at 35,000 mass resolution along with Waters ACQUITY ultra-performance liquid chromatography (UPLC) (Waters Corporation, Milford, MA, USA). A thorough explanation of the process has already been provided [18]. Hits were matched with pre-existing library entries of over 3300 pure standard chemicals to identify the compounds. Compounds were divided into several groups according to their sources. Internal standards and quality checks were previously published [19]. In short, to adjust for discrepancies in sample preparation and instrument performance, a combination of stable isotope-labeled chemicals was utilized as internal standards. The stability and repeatability of the procedure were tracked over time using quality control samples. To reduce variability and guarantee the integrity of the samples, a systematic methodology was employed for pre-analytical sample management, including sample collection, storage, and preparation. Raw metabolomic data underwent standardized preprocessing to ensure high quality and reliability. This included chromatographic peak detection, alignment, and deconvolution to obtain robust metabolite features. Metabolites with excessive missing data were excluded, while remaining missing values were imputed using established statistical approaches. To minimize batch effects, quality control (QC) samples were intermittently injected throughout the analytical runs to monitor instrument performance and allow correction for technical variation. Metabolomics data were log-transformed to ensure distribution normality. Batch correction was already performed by Metabolon by rescaling each metabolite so that its median is equal to 1. Metabolite data were further normalized relative to internal standards and total signal intensity to improve comparability across samples. Metabolites exhibiting high coefficient of variation (CV > 20%) across QC replicates were excluded to ensure analytical reproducibility. Sample injection order was randomized to prevent systematic run-order or batch bias.

### 2.3. Statistical Analysis

Clinical data were expressed as mean (standard deviation) or median (interquartile range), depending on distribution as assessed by the Shapiro–Wilk normality test. Comparisons between the control group and MASLD group were conducted using either Student’s *t*-test or the Mann–Whitney U test, as appropriate. A two-sided *p*-value < 0.05 was considered statistically significant. Metabolomics data were log transformed prior to analysis. Principal Component Analysis (PCA) was performed to evaluate the quality and variation in the data. The metabolic signatures of each group were distinguished using orthogonal partial least squares discriminant analysis (OPLS-DA) in SIMCA version 18. Model robustness was assessed using 7-fold cross validation and 200 permutation tests (default SIMCA settings) to prevent overfitting. Linear regression models were used for each metabolite, with the study group (Control/MASLD) as the primary independent variable and the metabolite level as the dependent variable. To account for any confounding, factors such as age, sex, BMI, and the first two principal components (PC1 and PC2) from the PCA were added in the model. False discovery rate (FDR) correction using the Benjamini–Hochberg (BH) method was applied to the regression results, and metabolites with FDR < 0.05 were considered significant and were selected for further analysis. Functional enrichment analysis was used to understand the metabolomic alterations’ biological significance using the Wilcoxon rank-sum test, and multiple testing correction was applied using the BH FDR method that led to the identification of enriched pathways. These sub-pathway classifications were provided by Metabolon’s reference library; no additional identifier mapping was required. Spearman’s correlation test was used to examine relationships between the significant metabolites and some clinical parameters for the entire cohort. All statistical analysis and figures were produced using R (4.2.1).

## 3. Results

### 3.1. General Characteristics of Participants

We employed propensity score matching techniques to balance the demographic characteristics of age, BMI, and sex between groups. As a result of this procedure, the final sample consisted of 110 participants, evenly distributed with 55 individuals in each group. There were no significant differences between the control and FLI groups in age, sex, or BMI (all *p* > 0.05). The MASLD group (Table 1) showed significantly higher glycemia, dyslipidemia, and elevated liver enzymes, along with lower HDL and SHBG levels.

### 3.2. Multivariate Analysis

**Figure 1:**
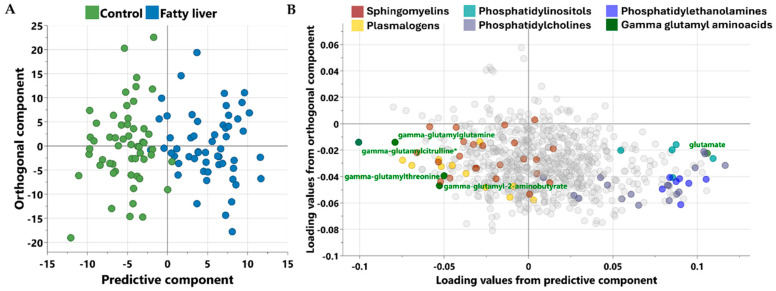
Scores plot (**A**) and loadings plot (**B**) from OPLS-DA between control (*n* = 55) and fatty liver participants (*n* = 55) [R^2^Y = 0.746; Q^2^ = 0.529]. Metabolites in color are the key discriminators, while less influential metabolites are shown in gray to reduce visual noise. * indicates a compound that has not been officially confirmed based on a standard, but that Metabolon is confident in its identity.

A non-targeted metabolomics analysis was conducted to characterize the metabolic profiles of each group. Orthogonal partial least squares discriminant analysis (OPLS-DA) was applied to determine the components that best differentiated the two groups, as illustrated in Figure 1. The scatter plot in Figure 1A demonstrates a clear separation between the groups, while Figure 1B presents the corresponding loading plots, highlighting the key metabolites contributing to this distinction.

### 3.3. Univariate Analysis

Linear regression analysis was performed to determine the metabolites differentiating the MASLD group from the control group. The model also contained age, sex, BMI, and principal components 1 and 2 from the PCA. Table 2 shows the most FDR significant metabolites. Results revealed that glutamate, along with phosphatidylethanolamines, phosphatidylinositols, and phosphatidylcholines, was strongly enriched in MASLD, whereas plasmalogens, sphingomyelins, and several gamma-glutamyl amino acids consistently showed higher levels in the control group, highlighting contrasting metabolic reprogramming between disease and healthy states.

### 3.4. Functional Enrichment Analysis

The results of functional enrichment analysis (Table 3) indicated significant differences in six pathways namely plasmalogens, sphingomyelins, gamma-glutamyl amino acids, phosphatidylethanolamines, phosphatidylcholines, and phosphatidylinositols.

### 3.5. Association Between Metabolites Associated with MASLD and Clinical Traits

**Figure 2:**
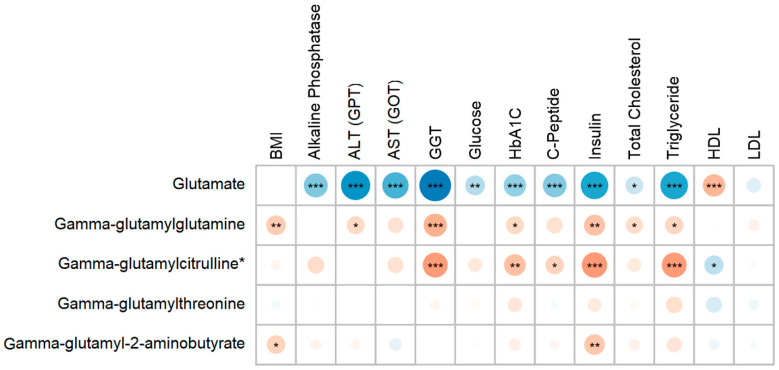
Correlation heatmap showing Spearman’s correlation between significant metabolites and key clinical parameters. The size of the circles within each cell indicates the magnitude of Spearman’s correlation coefficient. Blue coloration denotes positive associations, while red indicates negative associations. The color intensity reflects the strength of the correlation between each metabolite and clinical trait. Asterisks represent significance levels (* *p* < 0.05, ** *p* < 0.01, *** *p* < 0.001).

Spearman’s correlation analysis revealed that glutamate exhibits robust positive associations with elevations in liver enzymes (ALT, AST, GGT), markers of insulin resistance (C-peptide, insulin), and adverse lipid profiles (triglycerides, LDL), highlighting its role as a key indicator of hepatic and metabolic dysfunction characteristic of MASLD. In contrast, the gamma-glutamyl amino acids demonstrate correlations with lower levels of liver enzymes, insulin, and triglycerides, supporting their function as markers of favorable metabolic health and their predominance in the control group. To promote clarity and reduce complexity, only selected metabolites and clinical parameters were presented (Figure 2).

## 4. Discussion

MASLD represents a spectrum of liver pathology, ranging from simple steatosis to steatohepatitis, fibrosis, and cirrhosis. Its pathogenesis is linked to cardiometabolic disturbances, including insulin resistance and atherogenic dyslipidemia. In this study, we applied untargeted metabolomic profiling to serum samples from individuals with and without MASLD, classified by the Fatty Liver Index, with the goal of identifying characteristic metabolic signatures and pathways that may underlie disease presence and progression.

Expectedly, individuals in the MASLD group displayed significantly higher levels of ALT, AST, ALP, and GGT, reflecting ongoing hepatic injury, cholestasis, and oxidative stress. However, albumin and bilirubin levels remained within normal limits, indicating early to intermediate disease stages rather than advanced fibrosis or cirrhosis. In addition, patients in the MASLD group exhibited a distinctly atherogenic lipid profile characterized by significantly elevated concentrations of triglycerides, total cholesterol, and LDL-C, accompanied by reduced HDL-C levels. This pattern represents classic atherogenic dyslipidemia, a well-recognized metabolic disturbance strongly implicated in the cardiometabolic complications of MASLD [20]. Another anticipated finding was the significant reduction in SHBG levels among MASLD patients. This observation aligns with prior studies, which consistently report that low SHBG is closely associated with both the presence and severity of MASLD [21,22].

Interestingly, a consistent and highly significant lipidomic pattern in the MASLD group is the depletion of plasmalogens and sphingomyelins. This observation echoes and extends our previous findings [23], where depletion of these lipid classes was identified as a hallmark of insulin resistance as defined by the triglyceride-glucose index. In contrast, phosphatidylcholine, phosphatidylethanolamine, and phosphatidylinositol species were elevated in MASLD, pointing toward broader lipid remodeling events. The recurrent observation of reduced plasmalogen and sphingomyelin levels positions their depletion as a core feature of metabolic dysfunction. Plasmalogens, in particular, are ether phospholipids with strong antioxidant capacity [24], and their reduction suggests a loss of protective buffering against oxidative stress, one of the main drivers of MASLD progression. Similarly, sphingomyelin depletion implicates altered membrane dynamics and signaling disturbances [25], further contributing to metabolic dysfunction.

It is imperative to mention that in humans, previous evidence linking plasmalogens to MASLD has been limited to measurements of total plasmalogen levels. For instance, Puri et al. [26] reported that overall plasmalogen concentrations were significantly decreased in patients with NASH compared with healthy controls. In contrast, more detailed mechanistic insights have been derived from preclinical mice studies. Jang et al. [27] demonstrated that endogenous plasmalogens protect against hepatic steatosis and NASH by enhancing fatty acid oxidation through a PPARα-dependent mechanism, while Liu et al. [28] showed that plasmalogen supplementation can reduce hepatic steatosis in mouse models of obesity. Our study extends this body of evidence by demonstrating a remarkable depletion of specific plasmalogen species in humans with MASLD. This advances beyond prior bulk-level human measurements and provides molecular resolution that has thus far only been available in animal studies. While our findings cannot yet establish causality, they do suggest that the reduction in discrete plasmalogen species represents a consistent metabolic feature of MASLD and may contribute to loss of protective antioxidant and metabolic functions. Taken together, these observations begin to narrow the translational gap between mouse and human studies and underscore the need for further investigation into plasmalogen species-specific roles in MASLD pathogenesis and therapeutic targeting.

Our univariate analysis also identified glutamate as the top metabolite associated with the MASLD group. Indeed, several human studies [29,30] have shown that circulating glutamate levels are elevated in individuals with MASLD. Moreover, elevated plasma glutamate levels are associated with the development of diabetes [31], insulin resistance [32], and subclinical atherosclerosis [33]. Interestingly, the control group exhibited a distinct metabolic signature defined by elevated levels of gamma-glutamyl amino acids, potentially reflecting efficient and balanced glutathione cycle activity in the absence of hepatic steatosis. While high glutamate in MASLD is known, the simultaneous inverse correlation with gamma-glutamyl amino acids could be considered a novel finding. It strongly supports the hypothesis that the entire gamma-glutamyl cycle is dysregulated and shunted towards breakdown rather than balanced cycling. Supporting this interpretation, cysteine-glutathione disulfide was also markedly depleted in the MASLD group. As a key component of redox homeostasis [34], its loss indicates impaired antioxidant defenses, consistent with increased oxidative stress and clinically relevant outcomes. Moreover, our correlation analysis revealed a clear dichotomy in correlation patterns between glutamate, which was strongly positively associated with markers of liver injury, insulin resistance, and dyslipidemia characteristic of MASLD, and the gamma-glutamyl amino acids, which were predominantly linked to lower levels of these metabolic risk parameters, indicating healthier metabolic profiles.

Our study demonstrates that stratification by FLI reveals a contrasting metabolic signature: gamma-glutamyl amino acids predominating in low FLI and glutamate in high FLI, highlighting a previously unrecognized shift in the gamma-glutamyl cycle that may reflect the transition from metabolic flexibility to dysfunction in fatty liver.

It is important to clearly acknowledge several limitations of this study. First, the cross-sectional design inherently constrains causal inference, making it impossible to determine whether the observed metabolic alterations are primary drivers of MASLD or secondary consequences arising during disease progression. This limitation underscores the critical need for future longitudinal studies to establish temporal relationships and assess the potential of these metabolites as predictive biomarkers for MASLD onset and progression. Second, while our findings demonstrate robust associations, the relatively modest sample size limits statistical power and the generalizability of conclusions. Furthermore, the reliance on the Fatty Liver Index for disease stratification, rather than liver histology as the gold standard, introduces potential misclassification that may affect interpretation. Lastly, the cohort exclusively comprises Qatari nationals and long-term residents, restricting the ethnic and genetic diversity represented in the study population. This limits the generalizability of our findings to broader, multi-ethnic populations. To address these limitations, future research should incorporate larger, prospectively followed multi-ethnic cohorts, use integrative multi-omics approaches, and include mechanistic investigations in both human subjects and experimental models to validate the role of plasmalogens, sphingomyelins, and gamma-glutamyl cycle metabolites in MASLD pathogenesis.

## 5. Conclusions

This study highlights a novel dimension of MASLD-associated metabolic dysregulation, characterized by the consistent depletion of plasmalogens and sphingomyelins alongside elevated glutamate and reduced gamma-glutamyl amino acids. This opposing pattern within the glutamate–gamma-glutamyl axis suggests a broader reconfiguration of amino acid and glutathione metabolism beyond glutamate accumulation alone. Future mechanistic studies will be essential to determine whether these changes are causal drivers of disease progression or adaptive responses to hepatic fat accumulation. The depletion of plasmalogens and the disruption of the glutamate–gamma-glutamyl pathway stand out as central features of metabolic dysfunction in MASLD, with clear potential to inform biomarker discovery, disease classification, and the design of targeted therapeutic strategies.

## Figures and Tables

**Table 1:** Demographic characteristics of participants.

Test	Variable	Control Group (FLI < 30) *n* = 55	MASLD Group (FLI ≥ 60) *n* = 55	*p* Value
FLI		22.2 (18.8–25.7)	69.3 (62.4–84.1)	<0.001
General characteristics	Sex (M/F)	23/32	23/32	0.99
	Age	42.9 (11.8)	44.2 (10.3)	0.55
	BMI (kg/m^2^)	28.9 (26.2–30.9)	28.7 (27.6–30.4)	0.781
	Waist to hip ratio	0.81 (0.08)	0.89 (0.09)	<0.001
	Systolic blood pressure (mmHg)	109 (101–116)	120 (112–132)	<0.001
	Diastolic blood pressure (mmHg)	73 (67–78)	78 (73–86.5)	0.001
	Handgrip left	28.5 (22–37.7)	28 (20–39)	0.599
	Handgrip right	30.5 (22–42)	32 (24–40)	0.972
Blood sugar	Fasting blood glucose (mmol/L)	4.9 (4.74–5.3)	5.4 (4.8–6.2)	0.016
	HbA1C (%)	5.45 (5.3–5.7)	5.7 (5.4–6.32)	0.016
	C-peptide (ng/mL)	1.9 (1.51–2.53)	2.74 (2.1–4.03)	<0.001
	Insulin (uU/mL)	8 (5–9)	14.2 (9–20)	<0.001
Lipid profile	Total cholesterol (mmol/L)	4.68 (1.06)	5.4 (0.97)	<0.001
	HDL-cholesterol (mmol/L)	1.49 (0.42)	1.15 (0.27)	<0.001
	LDL-cholesterol (mmol/L)	2.71 (0.94)	3.2 (1.03)	0.011
	Triglyceride (mmol/L)	0.83 (0.64–1.25)	2.18 (1.51–2.86)	<0.001
Cardiac function	NT-proBNP (pg/mL)	33 (18.5–57.5)	22.5 (11.8–43.7)	0.097
	Homocysteine (µmol/L)	8 (6.95–10.1)	8.1 (6.9–9.4)	0.477
Kidney function	Creatinine (µmol/L)	66.16 (16.21)	65.44 (12.94)	0.910
	Urea (mmol/L)	4.4 (3.4–5.3)	4.3 (3.5–5)	0.560
	Bicarbonate (mmol/L)	27 (26–29)	27 (25–28)	0.051
	Total protein (g/L)	71.25 (4.18)	74.06 (3.37)	<0.001
Liver function	Alkaline phosphatase (U/L)	59 (50.5–72)	74 (63.5–85)	<0.001
	ALT (U/L)	16 (11.5–21)	32 (21–40.5)	<0.001
	AST (U/L)	16 (13–19)	23 (18–31)	<0.001
	GGT (U/L)	12 (8.5–15)	44 (24.5–84)	<0.001
	Albumin (g/L)	44.96 (2.45)	45.31 (3.14)	0.521
	Bilirubin (µmol/L)	6.9 (4.5–8)	6 (4–7)	0.209
Hormones	SHBG (nmol/L)	54 (35.5–78.75)	31 (21–42)	<0.001
	T4 (pmol/L)	13.4 (12.35–14.1)	13.2 (12.25–14.47)	0.901
	T3 (pmol/L)	4.33 (0.53)	4.52 (0.59)	0.097

Data are expressed as mean (standard deviation) or median (interquartile range), depending on the distribution assessed using the Shapiro–Wilk normality test. Comparisons between the control and fatty liver groups were conducted using either Student’s *t*-test or the Mann–Whitney U test, as appropriate. A *p*-value < 0.05 was considered statistically significant. Abbreviations: FLI, Fatty Liver index; BMI, Body mass index; HbA1C, glycated hemoglobin; HDL, high-density lipo-protein; LDL, low-density lipoprotein; NT-proBNP, N-terminal pro–B-type natriuretic peptide; ALT, alanine transaminase; AST, aspartate aminotransferase; GGT, Gamma-glutamyl transferase; SHBG, sex hormone-binding globulin; T4, Thyroxine; T3, triiodothyronine.

**Table 2:** Linear regression analysis was performed to determine the metabolites associated with the MASLD group. The model also contained age, sex, BMI, and principal components 1 and 2 from the PCA. * indicates a compound that has not been officially confirmed based on a standard, but that Metabolon is confident in its identity.

Metabolites	Super-Pathway	Sub-Pathway	Estimate	SE	*p*-Value	FDR
Glutamate	Amino Acid	Glutamate Metabolism	0.537	0.069	4.4 × 10^−12^	3.6 × 10^−9^
1-(1-enyl-palmitoyl)-2-oleoyl-GPC (P-16:0/18:1) *	Lipid	Plasmalogen	−0.379	0.056	1.1 × 10^−9^	4.4 × 10^−7^
1-palmitoyl-2-palmitoleoyl-GPC (16:0/16:1) *	Lipid	Phosphatidylcholine	0.496	0.083	2.9 × 10^−8^	6.1 × 10^−6^
Gamma-glutamylcitrulline *	Peptide	Gamma-glutamyl Amino Acid	−0.524	0.089	5.8 × 10^−8^	8.0 × 10^−6^
1-(1-enyl-palmitoyl)-2-linoleoyl-GPC (P-16:0/18:2) *	Lipid	Plasmalogen	−0.369	0.064	9.8 × 10^−8^	1.2 × 10^−5^
Sphingomyelin (d18:2/24:1, d18:1/24:2) *	Lipid	Sphingomyelins	−0.240	0.045	6.6 × 10^−7^	6.8 × 10^−5^
1-(1-enyl-palmitoyl)-2-palmitoyl-GPC (P-16:0/16:0) *	Lipid	Plasmalogen	−0.243	0.047	1.2 × 10^−6^	1.1 × 10^−4^
1-palmitoyl-2-oleoyl-GPE (16:0/18:1)	Lipid	Phosphatidylethanolamine	0.511	0.100	1.4 × 10^−6^	1.2 × 10^−4^
Sphingomyelin (d18:1/24:1, d18:2/24:0) *	Lipid	Sphingomyelins	−0.200	0.042	6.5 × 10^−6^	3.8 × 10^−4^
Cysteine-glutathione disulfide	Amino Acid	Glutathione Metabolism	−0.760	0.172	9.1 × 10^−6^	4.4 × 10^−4^
1-palmitoyl-2-arachidonoyl-GPI (16:0/20:4) *	Lipid	Phosphatidylinositol	0.370	0.080	1.3 × 10^−5^	5.9 × 10^−4^
1-palmitoyl-2-linoleoyl-GPE (16:0/18:2)	Lipid	Phosphatidylethanolamine	0.424	0.093	1.5 × 10^−5^	6.6 × 10^−4^
1-palmitoyl-2-arachidonoyl-GPE (16:0/20:4) *	Lipid	Phosphatidylethanolamine	0.404	0.091	2.2 × 10^−5^	8.9 × 10^−4^
1-(1-enyl-palmitoyl)-2-palmitoleoyl-GPC (P-16:0/16:1) *	Lipid	Plasmalogen	−0.330	0.075	2.8 × 10^−5^	1.0 × 10^−3^
1-myristoyl-2-arachidonoyl-GPC (14:0/20:4) *	Lipid	Phosphatidylcholine	0.473	0.109	3.4 × 10^−5^	1.1 × 10^−3^
1-myristoyl-2-palmitoyl-GPC (14:0/16:0)	Lipid	Phosphatidylcholine	0.451	0.104	3.5 × 10^−5^	1.1 × 10^−3^
Gamma-glutamylglutamine	Peptide	Gamma-glutamyl Amino Acid	−0.287	0.067	4.3 × 10^−5^	1.2 × 10^−3^
Palmitoyl sphingomyelin (d18:1/16:0)	Lipid	Sphingomyelins	−0.143	0.035	8.4 × 10^−5^	2.1 × 10^−3^
Serine	Amino Acid	Glycine, Serine and Threonine Metabolism	−0.150	0.038	1.2 × 10^−4^	2.9 × 10^−3^
1-palmitoyl-2-docosahexaenoyl-GPE (16:0/22:6) *	Lipid	Phosphatidylethanolamine	0.472	0.119	1.3 × 10^−4^	3.1 × 10^−3^
1-palmitoyl-2-linoleoyl-GPI (16:0/18:2)	Lipid	Phosphatidylinositol	0.311	0.081	2.0 × 10^−4^	4.6 × 10^−3^
1-palmitoyl-2-dihomo-linolenoyl-GPC (16:0/20:3n3 or 6) *	Lipid	Phosphatidylcholine	0.222	0.058	2.3 × 10^−4^	4.9 × 10^−3^
Gamma-glutamyl-2-aminobutyrate	Peptide	Gamma-glutamyl Amino Acid	−0.307	0.081	2.7 × 10^−4^	5.7 × 10^−3^
1-stearoyl-2-docosahexaenoyl-GPC (18:0/22:6)	Lipid	Phosphatidylcholine	0.301	0.081	3.1 × 10^−4^	6.0 × 10^−3^
Sphingomyelin (d18:1/22:1, d18:2/22:0, d16:1/24:1) *	Lipid	Sphingomyelins	−0.133	0.036	3.3 × 10^−4^	6.3 × 10^−3^
1-stearoyl-2-oleoyl-GPE (18:0/18:1)	Lipid	Phosphatidylethanolamine	0.368	0.099	3.5 × 10^−4^	6.3 × 10^−3^
Sphingomyelin (d18:2/18:1) *	Lipid	Sphingomyelins	−0.194	0.053	3.6 × 10^−4^	6.4 × 10^−3^
1-stearoyl-2-linoleoyl-GPE (18:0/18:2) *	Lipid	Phosphatidylethanolamine	0.352	0.097	4.5 × 10^−4^	6.8 × 10^−3^
Sphingomyelin (d18:2/24:2) *	Lipid	Sphingomyelins	−0.215	0.060	4.9 × 10^−4^	7.3 × 10^−3^
Gamma-glutamylthreonine	Peptide	Gamma-glutamyl Amino Acid	−0.213	0.061	7.8 × 10^−4^	1.1 × 10^−2^
Sphingomyelin (d18:2/16:0, d18:1/16:1) *	Lipid	Sphingomyelins	−0.126	0.036	8.9 × 10^−4^	1.2 × 10^−2^
1-(1-enyl-palmitoyl)-2-oleoyl-GPE (P-16:0/18:1) *	Lipid	Plasmalogen	−0.193	0.057	1.1 × 10^−3^	1.4 × 10^−2^
1-(1-enyl-palmitoyl)-2-linoleoyl-GPE (P-16:0/18:2) *	Lipid	Plasmalogen	−0.275	0.082	1.2 × 10^−3^	1.4 × 10^−2^

**Table 3:** Functional enrichment analysis of significantly altered metabolites (FDR < 0.05) based on sub-pathway classification. Wilcoxon sum of ranks test was used to identify overrepresented pathways among the differential metabolites.

Enriched Pathways	*p*-Value	FDR
Phosphatidylethanolamine	5.1 × 10^−7^	4.7 × 10^−5^
Sphingomyelins	5.0 × 10^−5^	2.3 × 10^−3^
Plasmalogen	1.0 × 10^−4^	3.1 × 10^−3^
Gamma-glutamyl amino acids	3.1 × 10^−4^	3.4 × 10^−3^
Phosphatidylcholine	5.4 × 10^−4^	1.2 × 10^−2^
Phosphatidylinositol	1.1 × 10^−3^	2.0 × 10^−2^

## Data Availability

The datasets used and/or analyzed during the current study are available from the corresponding author on reasonable request.
